# Addressing the Social Determinants of Health in the Amazon River Communities of Loreto, Peru, to Improve Maternal Health Access

**DOI:** 10.7759/cureus.82642

**Published:** 2025-04-20

**Authors:** Shanon Agbeve, Elizabeth R Burgess, Mahalah Joseph, Rosemary Wright, Rachel Clarke, Jessica B Oliveira

**Affiliations:** 1 Department of Medical Education, Florida International University, Herbert Wertheim College of Medicine, Miami, USA

**Keywords:** amazon river, maternal health, peru, rural health, social determinants of health

## Abstract

Background: Loreto, Peru, is a region marked by social inequalities, with about 55% of its one million inhabitants living in isolated river-edge communities. Indigenous populations face significant barriers to healthcare, education, and sanitation, resulting in high rates of infant mortality and maternal deaths. This study explores the demographic characteristics and social determinants of health in five communities along the Amazon River, with a focus on women’s access to healthcare. Understanding these barriers can help develop interventions specific to the needs of these communities.

Methods: Secondary data from a 2023 survey conducted by the Herbert Florida International University Wertheim College of Medicine medical students and Project Amazonas were analyzed. The study was conducted in five remote river communities within the Tamshiyacu Tahuayo Regional Conservation Area: El Chino, El Charro, Esperanza, Buena Vista, and Huaysi. Participants (n = 32) were Spanish-speaking women aged 18-80 years residing in these communities. They were randomly selected during medical visits, where they provided consent to participate. The survey was administered verbally in Spanish by Spanish-speaking medical students. Additionally, the Project Amazonas representative verbally asked a series of demographic questions in Spanish to the community leader in each village. Descriptive statistics were used to summarize demographic characteristics, general pregnancy and labor attitudes and experiences, and contraceptive use.

Results: The long travel distance via boat to healthcare facilities, poor infrastructure, and limited education all restrict access to maternal care. Only 42% of women received prenatal care at local health posts, and 40% delivered at home. Contraceptive use, particularly medroxyprogesterone acetate (Depo-Provera) injections, is prevalent at 71%, though unplanned pregnancies (28%) and child mortality remain common (19%).

Conclusions: Geographic isolation has a significant impact on healthcare access, with education and transportation emerging as critical factors in maternal health outcomes. The findings highlight the need for culturally sensitive interventions that address these root causes. Proposed sustainable solutions include basic medical training for community members, emergency plans, and improved transportation to reduce maternal health disparities in these communities.

## Introduction

Approximately 1,000,000 individuals live within Loreto, Peru’s most remote region, where the majority is covered by the Amazon Jungle [1]. Approximately 45% of the population resides in urban dwellings of Iquitos, while the remaining 55% inhabit approximately 2,000 remote, rural river-edge communities along the Amazon; these areas are secluded and only accessible by boat [2]. These indigenous communities suffer greater social inequalities, including reduced access to healthcare services, lower education level, fewer employment opportunities, declined nutritional status, and a greater infant mortality rate compared to the general population in Peru [2,3].

During 2020, there were 410 maternal deaths in Peru, with a maternal mortality ratio estimated at 69 maternal deaths per 100,000 live births [4]. Even though there has been a reduction in maternal mortality compared to previous years, deaths and complications related to pregnancy and childbirth continue to be a public health problem, characterized by unequal geographic and socioeconomic distribution [5]. According to the General Epidemiology Directorate of the Peruvian Ministry of Health, regions in Peru with the highest maternal mortality rates are Lima, La Libertad, Piura, Cajamarca, and Loreto [6].

The government-subsidized health insurance plan, Seguro Integral de Salud, was introduced in Peru in 2002 to provide free or low-cost health coverage to Peruvians living in poverty, particularly women and children, thereby helping to overcome barriers to healthcare access [7]. However, these interventions have largely focused on more populated urban areas, leading to a more significant reduction in maternal mortality in cities compared to rural regions. This disparity highlights the unequal distribution of health resources, further exacerbating social inequalities, particularly in remote communities like those along the Amazon [8].

Many social barriers are observed among families living in river-edge communities, including distance to healthcare facilities, lack of money and transportation, reluctance to travel alone, and women requiring permission to seek care from their partners [7,9]. Indications of poverty within the Peruvian Amazon communities include overcrowded households, poor sanitation, and food insecurity [8]. This severe inequity leaves many of the poorest women without access to life-saving maternal health services. Maternal mortality could be greatly reduced if these barriers that prevent or delay women from accessing routine and emergency obstetric services were addressed.

Certain social determinants of health (SDOH) have been studied within Peruvian Amazon river communities, though much of the research remains limited. Project Amazonas, a Peruvian-USA nonprofit, nonsectarian, and nonpolitical organization, has contributed to humanitarian, conservation, education, and research efforts in this region [2]. However, there is a notable gap in the literature specifically examining how SDOH influences maternal health in these remote communities.

In collaboration with Florida International University medical students and Project Amazonas, this study sought to identify key SDOH impacting women's health during a medical outreach intervention in five river communities of the Tamshiyacu Tahuayo Regional Conservation Area (ACRCTT) [2]. The objectives were as follows: 1) to examine SDOH within these communities and 2) to investigate women's accessibility to and utilization of healthcare services, including the cultural and social influences shaping maternal health practices. By better understanding the specific SDOH that influences maternal health outcomes, this study provides a foundation for the future development of long-term, sustainable interventions aimed at improving maternal care in these underserved communities. Ultimately, addressing these factors will help guide culturally sensitive solutions that target the root causes of maternal health disparities.

## Materials and methods

Setting

**Figure 1:**
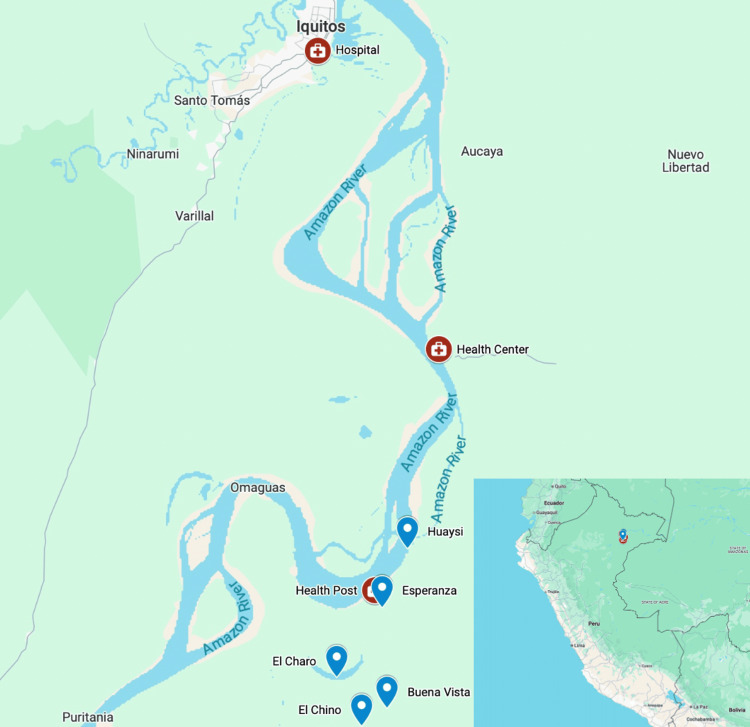
Map of the five communities (El Charro, El Chino, Esperanza, Buena Vista, and Huaysi) and medical buildings (Health Post in Esperanza, Health Center in Tamshiyacu, Hospital in Iquitos) along the Tahuayo River

The study was conducted in the Tamshiyacu Tahuayo Regional Conservation Area (ACRCTT). This is a protected area located southeast of Iquitos, extending over the Peruvian department of Loreto [10]. The Tamshiyacu Tahuayo Communal ACRCTT, which protects 420,080 hectares of forest, was established on May 16, 2009, as a result of the collaboration between the 35 communities that inhabit its zone [10]. The Tahuayo River runs approximately 80 km and is characterized as being acidic and nutrient-poor [11]. The communities along the river use the water from this river for drinking, fishing, cleaning, and bathing. This study focused on five river communities in the conservation area along the Tahuayo River: El Chino, El Charro, Esperanza, Buena Vista, and Huaysi (Figure 1).

Participants

There were two groups of participants. Participants in the survey were women between the ages of 18 and 80 years living in one of the five River Tahuayo communities, Spanish-speaking, and willing and able to provide verbal consent. We recruited the participants from a location where Project Amazonas created a temporary clinic within each community. Women who visited but did not reside in these communities and who were unable to speak Spanish were excluded from this study.

In addition, community leaders previously identified by Project Amazonas, who have a rich knowledge of their communities, were included in the study.

Study design

The study is a secondary analysis of qualitative and quantitative survey data collected in 2023 by medical student volunteers from Florida International University Herbert Wertheim College of Medicine (FIU HWCOM), working with Project Amazonas, a Peruvian-USA nonprofit organization dedicated to humanitarian, conservation, education, and research activities in the Peruvian Amazon. The organization has been working with the communities of ACRCTT for over 30 years.

Data collection

This research study used a secondary dataset from two sources: 1) a survey administered to women living in the five river communities: El Chino, El Charro, Esperanza, Buena Vista, and Huaysi, and 2) one-on-one interviews with community leaders.

A 20-question survey about maternal health outcomes was administered to women participants. Surveys were conducted by four Spanish-speaking medical students who were involved with Project Amazonas. An elementary level of Spanish was used to ensure that participants understood each question. A convenience sample was used to collect the answers. Interviewers from Project Amazonas read all 20 questions aloud to the participants and filled out the surveys in or near the building where medical care was provided for that community. All questions focused on birthing and prenatal care practices, family planning, and healthcare access. The surveys were conducted verbally with responses recorded on paper due to a lack of internet access. The survey consisted of 18 multiple-choice questions and two open-ended questions. None of the questions were validated. The interviews took around 5-10 minutes to complete.

In addition, a group interview consisting of 11 community leaders previously identified by Project Amazonas and who have a rich knowledge of their communities was implemented. The community leaders' group interview lasted around 30 minutes and was semistructured. Around 10 questions were asked, and community leaders were able to add additional questions and make comments not related to the previously prepared questions. Community leaders were asked about SDOHs, including the community population (i.e. number of families living in the community), internet and phone access, health care access (i.e., hospital, health post, and health center), transportation access, electricity access, water access, education access (i.e., types of schools), source of employment/income, and major health issues.

Data analysis

Quantitative data were analyzed using the Statistical Package for the Social Sciences version 28 (IBM Corp., Armonk, NY). Descriptive analyses, including univariate and bivariate frequencies, means, and standard deviations, were used to describe the study population, as well as to describe the knowledge and practices of participants. Qualitative data were analyzed using content analysis to identify key patterns and themes.

Ethical approval

The research study received exemption from the Institutional Review Board at Florida International University.

## Results

The SDOH assessment for the five river communities is summarized in Table 1. Across these communities, the primary sources of livelihood were agriculture and fishing. Access to essential services such as healthcare and education varied. Only Esperanza and El Chino had both primary and secondary schools. Access to healthcare facilities, especially hospitals, was limited due to significant travel times by speedboat. Only one community, Esperanza, had a designated health post that was recently upgraded to a level one hospital by the Ministry of Health.

**Table 1:** Social determinants of health information of El Charro, El Chino, Esperanza, Buena Vista, and Huaysi ^*^Health post: small local clinic run by technicians ^†^Health center: clinic aimed at providing general medical care ^‡^Hospital: provides more complex medical care, speedboat from Tamshiyacu costs s/25 (6.75 USD) GI: gastrointestinal

Community	El Chino	El Charro	Esperanza	Buena Vista	Huaysi
Population	60 families	16 families	40 families	17 families	12 families
Wi-Fi /Phone	No cell signal, but one person has Starlink and charges s/2.5 per 30 minutes of internet	No public phone or cell phone signal. The Movistar signal can be obtained with a fixed phone at times	There is no public phone or Wi-Fi, but the clinic has Wi-Fi	There is no public phone or cell signal. The police post has a satellite phone	No public phone, but there is good Movistar reception
Healthcare	Closest health post^*^ = Esperanza (60 minutes by small boat); closest health center^†^ = Tamshiyacu (3 hours by small boat); closest hospital^‡^ = Iquitos (90 minutes by speedboat from Tamshiyacu)	Closest health post^*^ = Esperanza (40 minutes by small boat); closest health center^†^ = Tamshiyacu (unknown boat time); closest hospital^‡^ = Iquitos (90 minutes by speedboat from Tamshiyacu)	Health post^*^ = within community (Staffed with nurses, two technicians, and maintenance personnel)	Closest health post^*^ = Esperanza (60-80 minutes by small boat); closest health center^†^= Tamshiyacu (120 minutes by small boat); closest hospital^‡^ = Iquitos (90 minutes by speedboat)	Closest health post^*^ = Nuevo Valentine (30 minutes by boat); closest health center^†^ = Tamshiyacu (2 hours by small boat); closest hospital^‡^ = Iquitos (90 minutes by speedboat from Tamshiyacu)
Common health problems	Respiratory tract infections, GI infections, snakebites	Respiratory tract infections, GI infections	Respiratory tract infections, GI infections	Respiratory tract infections, GI infections	Not specified
Transportation	Boat only	Boat only	Boat only	Boat only	Boat only
Electricity	Solar panels in school and homes: nonfunctioning due to a lack of maintenance	Half the houses have solar panels, but there is no solar in the school or the school kitchen.	Solar panels are at the clinic, the primary school, and the secondary school. Most houses have solar panels	There are solar panels in most houses and the primary school; most are nonfunctional	No solar panels, no electricity
Water	No potable water, source of water from river	No potable water, source of water from river	Newly built community water system. The schools have a rainwater catchment system with holding tanks.	No potable water, source of water from river	No potable water, source of water from river
Education	Kindergarten, primary school, and secondary school	Primary school only	Kindergarten, primary school, and secondary school	Primary school only	Primary school only
Economy	Fishing, agriculture, and handicrafts	Fishing and agriculture	Fishing, agriculture. Training center to teach sewing, fabric painting, cosmetology, and hair cutting	Fishing and agriculture	Fishing, agriculture, and handicrafts
Priorities	Medical kit, health promoter, reliable cellular	Medical kit, solar panel maintenance, and reliable cellular	Reliable cellular	Medical kit, small boat, primary school teacher	Medical kit, garbage management, and reliable cellular

Community leaders in the five river communities defined population by the number of families rather than the number of individuals. This distinction was noted across all five river communities. Among the five river communities surveyed, there were 145 families identified. El Chino and Esperanza stood out with the largest number of families, comprising 60 and 40 families, respectively. This unique method of population estimation by families rather than individuals reflects local cultural norms and community structure, highlighting an important aspect of social organization within these river communities.

Access to potable water was limited; Esperanza was the only community with an established water system. Most communities relied on river water for drinking, fishing, bathing, cleaning, and cooking. Access to electricity and communication infrastructure, such as Wi-Fi and cellular networks, was also limited across all communities.

The most commonly reported health issues among these communities included respiratory tract infections and gastrointestinal infections. Community leaders identified medical kits and reliable cellular communication as top health priorities.

A survey about maternal health outcomes was administered to 32 participants. Table 2 shows the demographic characteristics of the women who were surveyed. The average age of women interviewed was 38.6 years. Just over half of the women (53%) reported that they had their first child between the ages of 15 and 17. The size of each woman’s family varied, with the largest proportion having three to five children. There was roughly an equal split between married and single respondents. From the five communities, a majority of the women (59%) had a primary school level of education only.

**Table 2:** Demographics of survey participants in El Charro, El Chino, Esperanza, Buena Vista, and Huaysi (n = 32)

Characteristic	Value, n (%)
Average age	38.6
Age at first birth	
15-17	17 (53%)
18-20	12 (38%)
21-25	3 (9%)
Number of children	
1-2	10 (31%)
3-5	13 (41%)
6-8	3 (9%)
9-10	4 (13%)
More than 10	1 (3%)
Unspecified	1 (3%)
Marital status	
Married	13 (41%)
Single	13 (41%)
Living with a partner	3 (9%)
Unspecified	3 (9%)
Highest level of education	
Less than primary	1 (3%)
Primary	19 (59%)
Some secondary	3 (9%)
Secondary	8 (25%)
University	1 (3%)
Community	
Buena Vista	7 (22%)
El Chino	7 (22%)
Esperanza	7 (22%)
Huaysi	5 (16%)
El Charro	5 (16%)
Unspecified	1 (2%)

**Figure 2:**
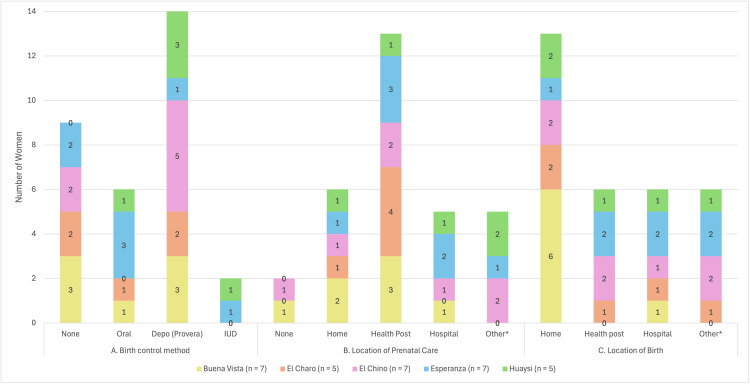
Survey responses. (A) Birth control method. (B) Location of prenatal care. (C) Location of birth ^*^These women reported giving birth to their children at multiple locations (combination of home, health post, and/or hospital) Only 31 participants responded instead of 32 IUD: intrauterine device

Table 3 summarizes pregnancy and labor attitudes and experiences among the 32 respondents. Results indicate that 69% used family planning methods, primarily medroxyprogesterone acetate (Depo-Provera) injections (Figure 2A). Additionally, 28% reported unplanned pregnancies, with 69% finding pregnancy and childbirth fearful. Around 19% experienced child deaths or complications within a month after delivery. All women breastfed their children, and 47% relied on others for health decisions, while only 3% reported healthcare staff discrimination or mistreatment.

**Table 3:** Attitudes and experiences about pregnancy and labor among women in the five river communities

Yes/no questions regarding pregnancy and the labor process	Value, n (%)
Ever used family planning methods	22 (69%)
Unplanned pregnancies	9 (28%)
Pregnancy/childbirth is a fearful time for respondents	22 (69%)
Experienced the death of a child	6 (19%)
Complication or death within the first month	6 (19%)
Breastfeed child	32 (100%)
Others help with making decisions about health	15 (47%)
Discrimination or mistreatment by healthcare staff	1 (3%)

Figure 2A illustrates contraceptive use by women in the communities. Medroxyprogesterone acetate (Depo-Provera) was the most common method overall, with usage rates varying across communities. In Esperanza, where the health clinic is located, women utilized multiple contraceptive forms (oral, Depo, and intrauterine device); two women reported no birth control use. Similarly, in Huaysi, the closest community to the health clinic, women employed various methods. Conversely, in El Chino, the farthest community from the clinic, Depo was most prevalent (71%), with some women using no contraception.

Figure 2B illustrates the distribution of prenatal care locations across the different communities. All women reported receiving prenatal care in El Charro, Esperanza, and Huaysi; these are the three communities closest to Esperanza’s health post. In all five communities, more women received prenatal care at a health facility than at home, with a higher proportion visiting the health post compared to the hospital. Despite 42% of women receiving care from the health post, the majority of women gave birth at home (Figure 2C). Over 40% gave birth at home, with the Buena Vista community having the highest percentage of home births. Eighty-seven percent of women in Buena Vista gave birth at home. The remaining communities demonstrated a uniform spread in birth locations, with one or two people giving birth at home, health post, hospital, or multiple places (Figure 2C). However, information on the presence or involvement of local midwives was not collected.

## Discussion

This study contributes to the existing literature by filling a critical gap in understanding the SDOH affecting maternal health in five remote river communities in the ACRCTT along the Amazon River. While there is some research on SDOH in Peru, this study specifically addresses the unique cultural and social factors influencing maternal health in this vulnerable region. By providing a detailed analysis of these determinants and their possible impact on healthcare access and utilization, the study offers valuable insights that can inform the development of targeted interventions. Issues such as water sanitation, education, transportation, electricity, and access to healthcare emerged as significant barriers that could impact health outcomes. This contribution helps to advance the knowledge base on maternal health disparities and supports the creation of effective strategies to improve health outcomes in these five underserved communities.

It has been shown that poor water sanitation is associated with an increased risk of diarrheal diseases and infant mortality [8]. Our findings indicated that access to potable water is very limited across all five communities. Esperanza was the only community with an established water system, constructed by Water Mission, a nonprofit organization based in the United States. The other communities were using water sourced directly from the nearby river. While we cannot definitively conclude a direct causal relationship between inadequate water sanitation and infant mortality, research shows that increasing access to water and sanitation are significantly associated with decreases in the negative health outcomes of interest, namely under-five child mortality, under-five child mortality due to diarrhea, infant mortality rate, and maternal mortality rate [12]. The impact of inadequate water sanitation and healthcare access may have partially contributed to the reports of child mortality and maternal complications after childbirth found within our study. Specifically, the study revealed that 19% of women reported the death of a child, while another 19% reported complications within the first month after childbirth.

Maternal education has also been shown to be an important social determinant of maternal and child health [13]. Schooling in itself has been a powerful tool to influence health; literate women tend to marry later and are more likely to use family planning methods [14]. Mothers with primary education tend to take better care of their children and are more likely to seek medical care, such as immunization, than those who lack schooling [14]. In 1993, Peru's education reform aimed to extend compulsory schooling in rural indigenous communities; however, enforcement challenges, insufficient public expenditure, and unaddressed preexisting disparities led to shortcomings [15]. Many children in rural areas have to travel long distances to schools and/or attend schools that lack well-trained teachers [16]. Some findings within our study may reflect deficiencies that the amended constitution faced in rural indigenous communities. We observed that all communities have primary schools, with only Esperanza and El Chino also having secondary schools. Additionally, 59% of women had primary education, while only 34% had some secondary education. Early childbirth rates and maternal complications could be influenced by educational attainment and cultural factors.

Access to health education, medical facilities, family planning, and childcare is crucial alongside formal schooling. Figure 2B reveals that most women (93%) received prenatal care at a health facility, with 42% using the health post in Esperanza, the nearest community. One explanation for the availability of prenatal care and family planning services is the partnership between the Ministry of Health and Angels of the Amazon (AoA), a nonprofit founded in 2007. AoA collaborated with the health post in Esperanza, which serves as the primary healthcare provider for residents of the Tahuayo River basin [17]. This partnership resulted in significant improvements to the clinic's infrastructure, staffing, and medical equipment, elevating it to a Level 1 hospital recognized by the Ministry of Health. AoA also supports health and education initiatives through workshops at the clinic and local schools, including a health and sex education program launched in May 2023 [17].

Nearly half of the women opted for home births, a trend particularly notable in Buena Vista, the farthest community from Esperanza (Figure 2C), possibly due to the fact that there is only one public boat that travels the river once a day. Transportation barriers, exacerbated by unreliable electricity, pose challenges to accessing healthcare facilities. Many travel in adverse weather conditions, sometimes at night, via slow boats or canoes [17]. This is a possible reason for the high percentage of home births seen in this study. In the remote river network of the Amazon, the distance from large cities and isolation helps to simultaneously protect the environment while also serving as a barrier to the social and economic development of its local communities [18]. Medroxyprogesterone acetate (Depo-Provera) was the predominant contraceptive method, utilized by nearly 64% of respondents (Figure 2A). Similarly, a study by Westgard et al. found that 63% of women in Loreto used contraception, with 43% opting for modern methods [13]. The reasons for its popularity, likely tied to accessibility and affordability, are complex to ascertain [19,20]. This higher adoption rate of modern contraceptives in our communities, compared to Loreto, could be linked to the presence of the AoA clinic in Esperanza, which would highlight the benefits of family planning and improved medication access facilitated by organizations like AoA.

With the information gathered, we plan to create tailored interventions for the communities studied. The initial priorities will focus on addressing transportation challenges, health education, and water hygiene, three important health priorities reported in the study. The plan includes identifying and facilitating the training of a community health worker (CHW), in addition to building health literacy among community members, beginning with discussions on water hygiene and sun safety. Moreover, medical students and faculty will collaborate with community leaders to develop emergency plans specific to each area.

Study limitations

This study utilized a convenience sample of 32 women, which is a small sample size and may limit the generalizability of its findings to the broader population of women in the assessed communities. Furthermore, the survey of women who sought care from the clinic introduces selection bias, as their characteristics and experiences may differ from those of women who did not seek care. Therefore, the findings may not fully represent the experiences of all women of the community. However, it begins to highlight some of the experiences of women in these locations.

Recall bias was apparent in many of the interviews. Most participants who were over the age of 60 years seemed to have trouble recalling their experiences with their pregnancies. Additionally, social desirability bias was observed in certain interviews. Several of the participants were shy and appeared to respond in a manner they thought was socially acceptable.

A language and cultural barrier also existed between the interviewers and the participants. All medical students who conducted the interview spoke Spanish. However, the medical students were not Peruvian and had limited cultural knowledge about the country. A local interpreter may have improved the rapport in interviews, but none was available. Despite the limitations, our study gives valuable insights into the SDOHs present and the future interventions needed.

Implications for policy and practice

This study, a collaboration between FIU HWCOM and Project Amazonas, established an ongoing partnership aimed at improving health access in river communities. Following the July 2023 trip, FIU HWCOM committed to returning annually to ensure continuity of care and education. The partnership's goal is to build a sustainable program that educates communities about their SDOH, thereby increasing knowledge and optimizing health access based on identified needs.

In July 2025, FIU HWCOM and Project Amazonas aim to work with the five community leaders to identify and begin to train CHWs. Education will initially focus on basic medical techniques, such as using automatic blood pressure machines, glucometers, and digital thermometers, along with discussions on water hygiene and sun safety. The team will also collaborate with leaders to help establish emergency plans, addressing critical needs such as transportation to health facilities.

Annual visits will continue to expand the CHWs' skills with new medical topics and community health strategies based on evolving needs. These efforts, guided by feedback and data collected during each visit, will foster a sustainable cycle of learning and improvement. The partnership's long-term goal is to enhance health access, outcomes, and resilience within these communities by ensuring interventions remain responsive to their changing health landscape.

Beyond healthcare education, the study's findings highlight additional challenges, including limited transportation, cell phone access, and access to potable water. Addressing these issues may require broader collaboration with local stakeholders, government agencies, and nonprofit organizations such as AoA.

## Conclusions

Surveying community leaders and women within five Amazon River communities in the ACRCTT was the initial step in understanding the complex factors that potentially influence maternal health disparities in this population. Our study revealed an interplay of socioeconomic factors, educational limitations, and geographical barriers that possibly contribute to the distinct health challenges faced by women in these communities. Health challenges observed in these communities can be broadly categorized into three areas: attitudinal and behavioral factors, such as apprehensions regarding childbirth and preferences for birthing outside healthcare facilities; physiological health issues, including gastrointestinal and respiratory infections, early-age pregnancies, and high rates of child mortality and morbidity; and SDOH, including water sanitation issues, electricity instability, limited access to education, and transportation barriers. These intersecting factors collectively contribute to the unique health disparities faced by women in these communities. While the Peruvian government has attempted to address some factors, such as mandating secondary education, limited resources have hindered its effectiveness. The presence of the nonprofit organization AoA in these communities has enhanced access to primary medical care, family planning, prenatal care, and health education. However, transportation barriers continue to pose challenges to accessing these services, highlighting the ongoing need for intervention and support.

Our approach, focusing on needs assessments and directly responding to community-specific challenges, can serve as a valuable model for mission-trip-based international programs. By prioritizing collaboration with local communities and being adaptable to their unique needs, we aim not only to address immediate health concerns but also to empower these populations for long-term improvement. This method fosters sustainable partnerships, enhances the effectiveness of interventions, and strengthens community resilience. Through continued engagement and adaptation, our goal is to create meaningful and sustainable change that can be replicated in similar contexts, thereby improving health outcomes and overall well-being in underserved communities.
